# Evaluation of brucellosis eradication strategies in water buffalo in a key dairy production area of southern Italy

**DOI:** 10.3389/fmicb.2026.1741007

**Published:** 2026-02-03

**Authors:** Celestina Mascolo, Alessandra Mazzeo, Lucia Maiuro, Simona Signoriello, Carlo Ferrara, Marco Esposito, Sebastiano Rosati, Elena Sorrentino

**Affiliations:** 1 Department of Prevention, Complex Structure Animal Health, Local Health Agency of Caserta, Caserta, Italy; 2 General Directorate for Health Protection and Coordination of the Regional Health System, Unit of Prevention and Veterinary Public Health, Naples, Italy; 3 Department of Agricultural, Environmental and Food Sciences (DiAAA), University of Molise, Campobasso, Italy; 4 Department of Mental Health and Public Medicine, Section of Statistics, University of Campania Luigi Vanvitelli, Naples, Italy; 5 Department of Agricultural, Forestry and Food Sciences (DISAFA), University of Turin, Grugliasco, Italy

**Keywords:** brucellosis, epidemiology, one health, reinfection, stamping out, water buffalo

## Abstract

Brucellosis in water buffalo remains endemic in southern Italy, particularly in areas of the province of Caserta characterized by high animal density. This retrospective cohort study (2016–2021) assessed the effectiveness of stamping out (whole-herd depopulation) *versus* selective culling in counteracting brucellosis in water buffalo herds. Data from 222 outbreaks were analyzed using Cox regression, incorporating herd size, buffalo density, eradication method, and co-infection with *Mycobacterium bovis*. Stamping out reduced reinfection risk by 80% (HR = 0.2; *p* < 0.001), especially in municipalities exceeding 200 buffaloes/km^2^. Co-infection with *M. bovis* was not statistically associated with reinfection. These results indicate that control strategies should prioritize stamping out, coupled with reinforced structural and operational biosecurity measures, even in high-density settings, to achieve disease-free status. Integrated surveillance and education, in line with One Health principles, are essential for sustainable eradication and protection of the buffalo dairy sector.

## 1 Introduction

Water buffalo (*Bubalus bubalis*) farming represents a centuries-old tradition in southern Italy and a cornerstone of the regional economy, especially in the Campania region. The production of Mozzarella di Bufala Campana, a Protected Designation of Origin (PDO) cheese, involves thousands of workers and generates substantial economic returns, with the province of Caserta hosting the largest and most densely concentrated water buffalo population in Italy (Mazzeo et al., 2024; ISTAT, 2023). Bovine brucellosis, mainly caused by *Brucella abortus* and occasionally *B. melitensis*, is an important zoonosis affecting water buffalo among other species (De Massis et al., 2019; Orru et al., 2024; Parolini et al., 2024; Ruano et al., 2025). This disease induces reproductive losses, milk discard, and trade restrictions, with significant public health and economic implications (Corbel, 1997; Islam et al., 2023; EFSA and ECDC (European Food Safety Authority and European Centre for Disease Prevention and Control), 2024; Pappas et al., 2006). Globally, brucellosis remains endemic in several regions, including the Mediterranean basin, the Middle East, Africa, Asia, and Latin America, where eradication is often hampered by high animal density, complex production systems, and limited resources (Dadar et al., 2019). In Europe, although several Member States have achieved the Disease Free (DF) status from brucellosis—previously indicated as Officially Brucellosis Free (OBF)—the disease remains endemic in the Mediterranean basin and South-Eastern Europe. In these regions, ovine and caprine brucellosis caused by *Brucella melitensis* continues to circulate, favored by extensive husbandry systems, pasture-based management, and cross-border movements of small ruminants. In countries such as Italy, Greece, Portugal and several Balkan States, compulsory eradication programs are still in place and represent a relevant European model of One Health control measures.

Brucellosis is WOAH-listed (World Organisation for Animal Health (WOAH), 2024) and, under the European Union (EU) legislation [Regulation (EU) 2016/429, 2016], is subject to compulsory surveillance and eradication measures in Member States. Italy has implemented eradication programs for decades, with stricter regional regulations introduced for high-prevalence areas to achieve Officially Brucellosis-Free (OBF) status (Italian Ministry of Health, 1989; Italian Ministry of Health, 1994; Tittarelli et al., 2015), as well as a specific plan recently issued for the Campania region and co-funded by the EU Commission (Campania Region, 2022). Approximately 70% of Italian water buffalo heads are located in Campania, with over 60% concentrated in the province of Caserta. High animal density, contiguous farms, complex hydrographic networks, low-altitude terrain and frequent flooding, create environmental conditions that facilitate pathogen persistence and transmission (Campania Region, 2017; Guerriero et al., 2019; Mazzeo et al., 2025b).

Once introduced into the herd, brucellosis is transmitted via environmental contamination through animal secretions and natural reproduction (Guarino et al., 2001; Laine et al., 2023). Notably, *B. abortus* can survive for extended periods in the environment under favorable conditions (Borriello et al., 2013; Spickler, 2018).

Transmission of brucellosis to humans occurs mainly through the consumption of unpasteurized milk and dairy products (Dadar et al., 2019; Khurana et al., 2021; Wang et al., 2024), or through occupational exposure among farmers, veterinarians, slaughterhouse workers, and laboratory personnel (Corbel, 1997; Franco et al., 2007; Lounes et al., 2022; Dadar et al., 2023; Robi et al., 2023). Consequently, outbreaks of buffalo brucellosis affect animal health, food safety, human health, and the local economy (Guarino et al., 2001; Mazzeo et al., 2024; Mazzeo et al., 2025b). Food safety and occupational biosafety measures are therefore essential. Italy also applies a national traceability system for the buffalo supply chain, designed to monitor the production and movement of buffalo milk and dairy products and to strengthen food safety and transparency along the entire value chain (Cappelli et al., 2021). Notably, in farms that have not yet achieved Disease-Free Status (DFS), as defined in Regulation (EU) 2016/429 (2016), raw milk from buffaloes that test negative and show no clinical signs of brucellosis may still be used, provided that the competent authority authorizes mandatory heat treatment in accordance with Regulation (EC), 2004 853/2004 and ISO Standard 11816-1 [Commission Regulation (EC) No 1664/2006 of 6 November 2006, 2006].

In 2024, the Decree of the Italian Ministry of Health (2024) established a mandatory national program for the eradication of bovine brucellosis seeking to reach and/or preserve the DFS within the Italian territory in the aim for complete eradication by 2030. Control of brucellosis relies on testing and culling of seropositive animals, while whole-herd depopulation (stamping out) is used in specific epidemiological context (SANCO/6095/2009, 2009). Complementary measures, including thorough disinfection, strengthened biosecurity, and adequate waiting periods before restocking, are essential to prevent reinfection. Nevertheless, latent infections, especially in female calves exposed in utero or early after birth, may still compromise eradication efforts (Akhtar and Mirza, 1995; Dolan, 1980; EFSA Panel on Animal Health and Welfare (AHAW) et al., 2017). Recent surveillance in Caserta shows a decline in positive animals from 11,930 in 2020 to approximately 6,000 in 2023, although the endemic focus remains difficult to eliminate (Mazzeo et al., 2023; Mazzeo et al., 2025b). Additional factors, such as co-infection with *Mycobacterium bovis*, may influence transmission dynamics and immune responses. Recent studies from Brazil have shown heterogeneous spatiotemporal patterns of brucellosis and tuberculosis in water buffalo, suggesting that herds can act as overlapping reservoirs and complicate eradication efforts (Schwarz et al., 2021). These co-infections intensify clinical disease and increase mortality (Lee et al., 2023) and have motivated the development of an RB51 expressing protective antigens of *M. bovis* (Schurig et al., 2021).

Despite decades of control efforts based on test-and-slaughter, vaccination, and movement restrictions, brucellosis persists in many endemic settings, underscoring the need for control strategies tailored to different production systems and supported by comparative international evidence (Dean et al., 2012; Dadar et al., 2019).

Based on this background, the present study compares the effectiveness of stamping out *versus* selective culling in eradicating brucellosis from water buffalo herds in the province of Caserta. Using retrospective outbreak data from 2016 to 2021, we also investigated how herd size, local buffalo density, and co-infection with *M. bovis* influence reinfection risk. In addition, we compare these findings with data up to the end of 2024 to assess the impact of regional rules introduced in 2022. Through this data-driven evaluation, the study aims to contribute not only to the national debate but also to the broader international understanding of brucellosis eradication in high-density buffalo production systems.

## 2 Materials and methods

We monitored outbreaks of buffalo brucellosis over a six-year period prior to the adoption of the Campania Region Plan 2022 and categorized them into two groups: farms subjected to whole-herd depopulation (stamping out) and farms undergoing selective culling of seropositive animals. Brucellosis diagnosis was performed by official veterinary services and authorized laboratories using the Rose Bengal Test (RBT) as a screening assay and the Complement Fixation Test (CFT) as a confirmatory test, in accordance with the WOAH *Manual of Diagnostic Tests and Vaccines for Terrestrial Animals* (World Organisation for Animal Health (WOAH), 2022; Mazzeo et al., 2025b). In parallel, the diagnosis of co-infection with *Mycobacterium bovis* was performed through *intra vitam* tests, including the intradermal tuberculin test and the interferon-gamma release assay (IGRA), which assess cell-mediated immune responses. The intradermal test requires reading 72 h after inoculation, while IGRA is performed in authorized laboratories on heparinized blood samples incubated with tuberculin. At slaughterhouses, suspicious organs were subjected to post-mortem examination, PCR, and culture for molecular typing of *M. bovis*. These diagnostic approaches are used to confirm or exclude infection and to support molecular epidemiological investigations in water buffalo (Giovannozzi et al., 2025; Mazzeo et al., 2025b).

Stamping out (SANCO 6095/2009) refers to whole-herd depopulation, removal, and mandatory downtime with reinforced environmental sanitation and strict biosecurity before repopulation. Selective culling is limited to the removal of positive animals with prompt but partial herd retention [Regulation (EU), 2016/429; Campania Regional Decree 2022; Italian Ministry of Health Decree 2024].

In these two groups, we aimed to assess (i) the incidence of reinfection over time, (ii) the interval until reinfection occurred, and (iii) the spatial distribution of reinfected farms to determine whether specific areas were at a higher risk of reinfection.

The data used for this analysis were obtained from the Italian Ministry of Health *via* the National Veterinary Information System (VETINFO, 2025), covering the period from January 1, 2016, to December 31, 2021. The database included all registered water buffalo brucellosis outbreaks reported by the Local Veterinary Units (ASL) of the province of Caserta.

Based on this dataset, the following inclusion and exclusion criteria were applied. All confirmed outbreaks of brucellosis in water buffalo registered in VETINFO between 1 January, 2016 and 31 December 2021, were initially considered eligible. Outbreaks were included if they had a confirmed diagnosis, a clearly defined eradication outcome (stamping out or selective culling), and complete information on farm identification and key epidemiological dates (outbreak confirmation and extinction), allowing follow-up for the assessment of reinfection.

Outbreaks were excluded if the infection was still ongoing as of 31 December 2021, since reinfection could not have occurred within the observation period, or if essential data were missing or inconsistent. According to these criteria, 38 records were excluded because the outbreak was still active at the end of the study period.

The original dataset also included farms that experienced multiple reinfection events during the observation period, including 65 farms with a second reinfection and 10 farms with a third reinfection. To meet the assumptions of independence required for survival analysis and to ensure comparability across farms, only the first reinfection event per farm was retained for Kaplan–Meier and Cox regression analyses, while records referring to second and third reinfections were excluded.

When multiple records referred to the same outbreak event in the same farm, entries were cross-checked using farm identification codes and dates. Duplicate records were removed, and records with ambiguous or conflicting information were excluded when reliable classification was not possible.

We created a geospatial map to assess the distribution of farms subjected to stamping out or selective culling using QGIS (version 3.24.0 Tisler, Free Software Foundation, Inc., Boston, MA, USA).

The data included geographical coordinates, farm identification codes, and the eradication method used. The shapefile of the study area was downloaded from the ISTAT website (ISTAT, 2023). Excel datasheets were imported into QGIS and converted into data layers. Classification techniques (WGS84) were applied to generate maps showing the distribution of reinfection events and eradicated outbreaks.

The following variables were included in the statistical analysis for each outbreak:

- outbreak identification number;
- municipality;
- farm identification code;
- farm geographic coordinates;
- confirmation date of the outbreak;
- eradication date and reinfection date (if applicable);
- number of positive buffaloes per year and per farm;
- total number of animals present on farm at the outbreak onset;
- method of eradication (stamping out or selective culling of seropositive animals);
- coinfection with *Mycobacterium bovis*;
- date of restocking after stamping out.

The interpretation of hazard ratios for herd size is specified as being calculated per increments of 50 buffaloes at outbreak onset, according to the risk stratification model adopted in the present study.

Additional derived variables were calculated, including:

- farm downtime (number of days without animals after stamping out);
- buffalo density (total number of buffaloes/km^2^) per increment of 200 buffaloes in each municipality where outbreaks occurred;
- reinfection time, calculated as the time between outbreak extinction and the subsequent reinfection event in the same farm;
- farm closing time, calculated as the time between stamping out and restocking, subtracted from the reinfection time.

Finally, to assess the effect of the new regional and national eradication plans, results obtained for 2016–2021 were compared with the epidemiological pattern observed at the end of 2024 (VETINFO) following implementation of the Campania Region Decree (2022) and Italian Ministry of Health Decree (2024).

### 2.1 Statistical analysis

Continuous variables were reported as means ± standard deviations (SD), while categorical variables as frequencies and percentages (%). Comparison of continuous variables were performed using Student’s *t*-test, whereas categorical variables were compared using Pearson’s chi-square test. The reinfection rate was estimated using the Poisson test on the subset of farms with at least one reinfection event. Cumulative incidence of reinfection was calculated using the Kaplan–Meier survival analysis (1-survival probability) and differences between the two control strategies (stamping out vs. selective culling) were evaluated using a two-sided log-rank test. Factors associated with time to reinfection were initially tested using univariate Cox regression models. Significant covariates were then included in a multivariate Cox proportional hazards model. Model selection was based on Bayesian Information Criterion (BIC) (Schwarz, 1978) to balance goodness-of-fit and model parsimony. All analyses were performed in R version 4.1.0 (R Foundation for Statistical Computing, 2018, Vienna, Austria). Results are reported as estimates with corresponding 95% confidence intervals (CI).

## 3 Results

A total of 260 confirmed outbreaks of water buffalo brucellosis were reported in the province of Caserta during the study period. However, 38 outbreaks were excluded because the infection was still ongoing as of 31 December 2021, making it impossible to assess recurrence.

**Figure 1:**
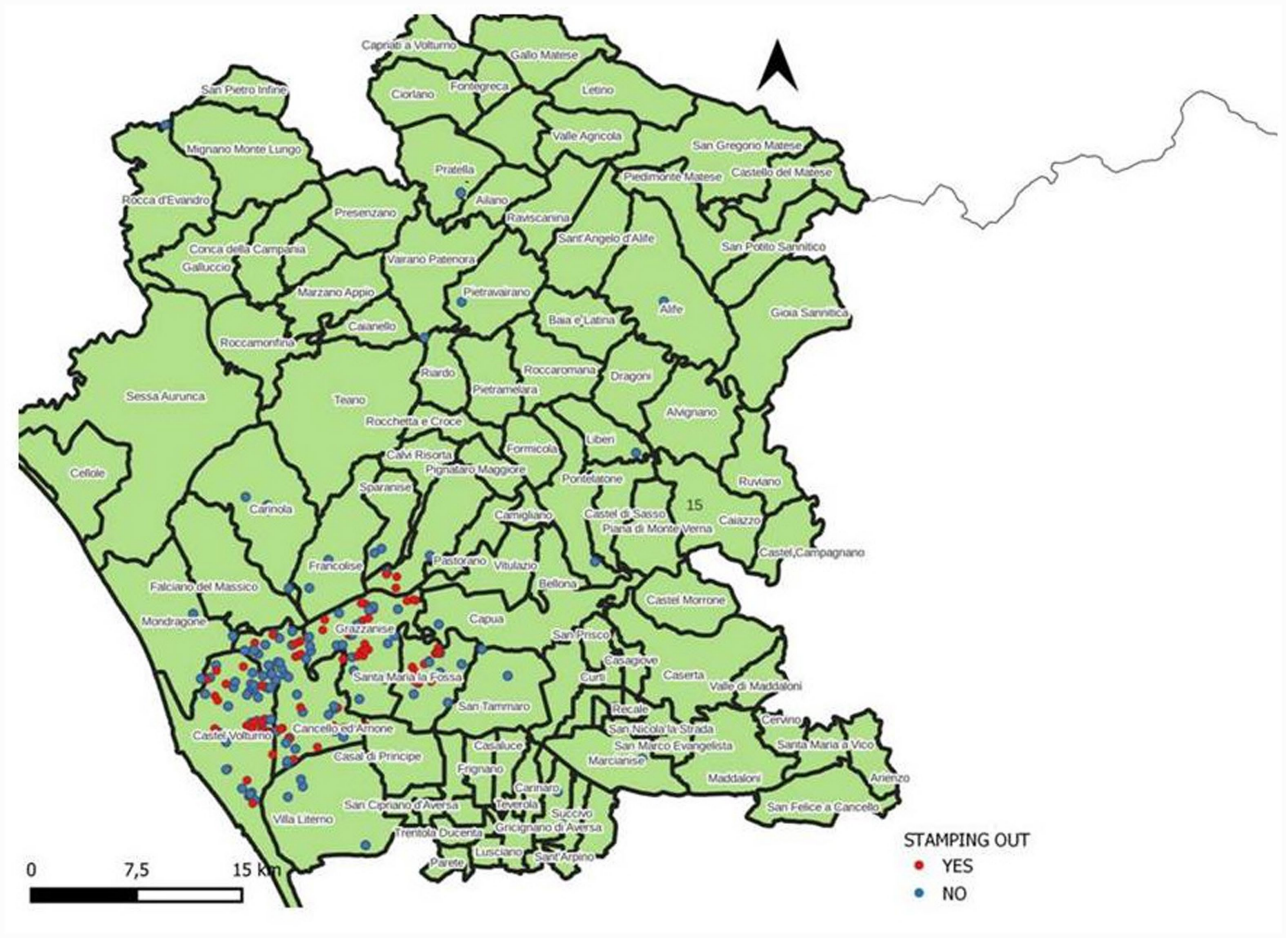
Distribution of buffalo brucellosis outbreaks in the province of Caserta between 01.01.2016 and 31.12.2021, according to control strategy: stamping out (red dots) and selective culling (blue dots).

**Figure 2:**
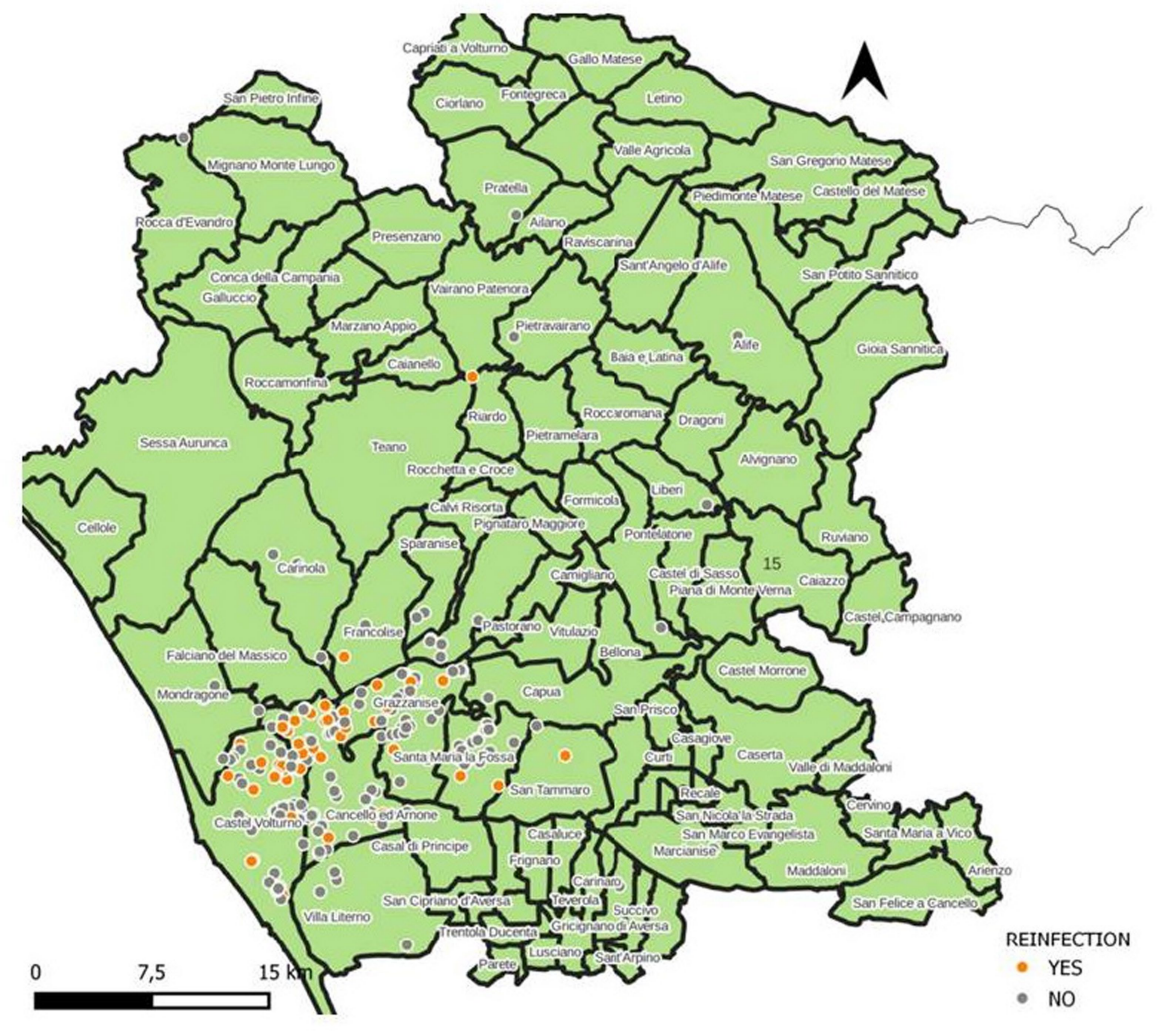
Distribution of farms with (orange dots) or without reinfection (grey dots) in the province of Caserta in the time period 01.01.2016–31.12.2021.

Spatial distribution of the stamping out procedures (red dots) and selective culling (blue dots) is shown in Figure 1. Spatial distribution of reinfection foci (orange dots) and farms where reinfection was not observed (grey dots) is shown in Figure 2.

Table 1 summarizes the characteristics of the 222 brucellosis outbreaks and the relationships between farms with (65 farms) and without (157 farms) reinfection.

**Table 1:** Characteristics of brucellosis outbreaks (*N* = 222) stratified by reinfection status.

VARIABLE	Absence of reinfection *N* = 157	Reinfection occurrence *N* = 65	*p*-value
Stamping out	78 (49.7%)	11 (16.9%)	<0.001
Selective culling	79 (50.3%)	54 (83.1%)	
Coinfection with *Mycobacterium bovis*	24 (15.3%)	7 (10.8%)	0.502
Absence of coinfection with *Mycobacterium bovis*	133 (84.7%)	58 (89.2%)	
Buffalo density: buffaloes/km^2^ (mean ± SD)	328 ± 164	361 ± 134	0.121
Number of buffaloes per farm at the outbreak onset (mean ± SD)	298 ± 200	389 ± 261	0.013

A significant association (*p* < 0.001) was found between reinfection occurrence and eradication strategy. Reinfection was observed in 54 out of 133 farms (40.7%) where outbreaks were controlled by selective culling, compared with only 11 out of 89 farms (12.4%) where stamping out was applied. Conversely, no significant association was observed between coinfection with *M. bovis* and reinfection (*p* = 0.50). Buffalo density across municipalities also showed no significant association with reinfection (*p* = 0.12). However, farms with reinfection had a significantly higher mean herd size at the outbreak onset compared with non-reinfected farms (389 ± 261 vs. 298 ± 200; *p* = 0.013).

**Figure 3:**
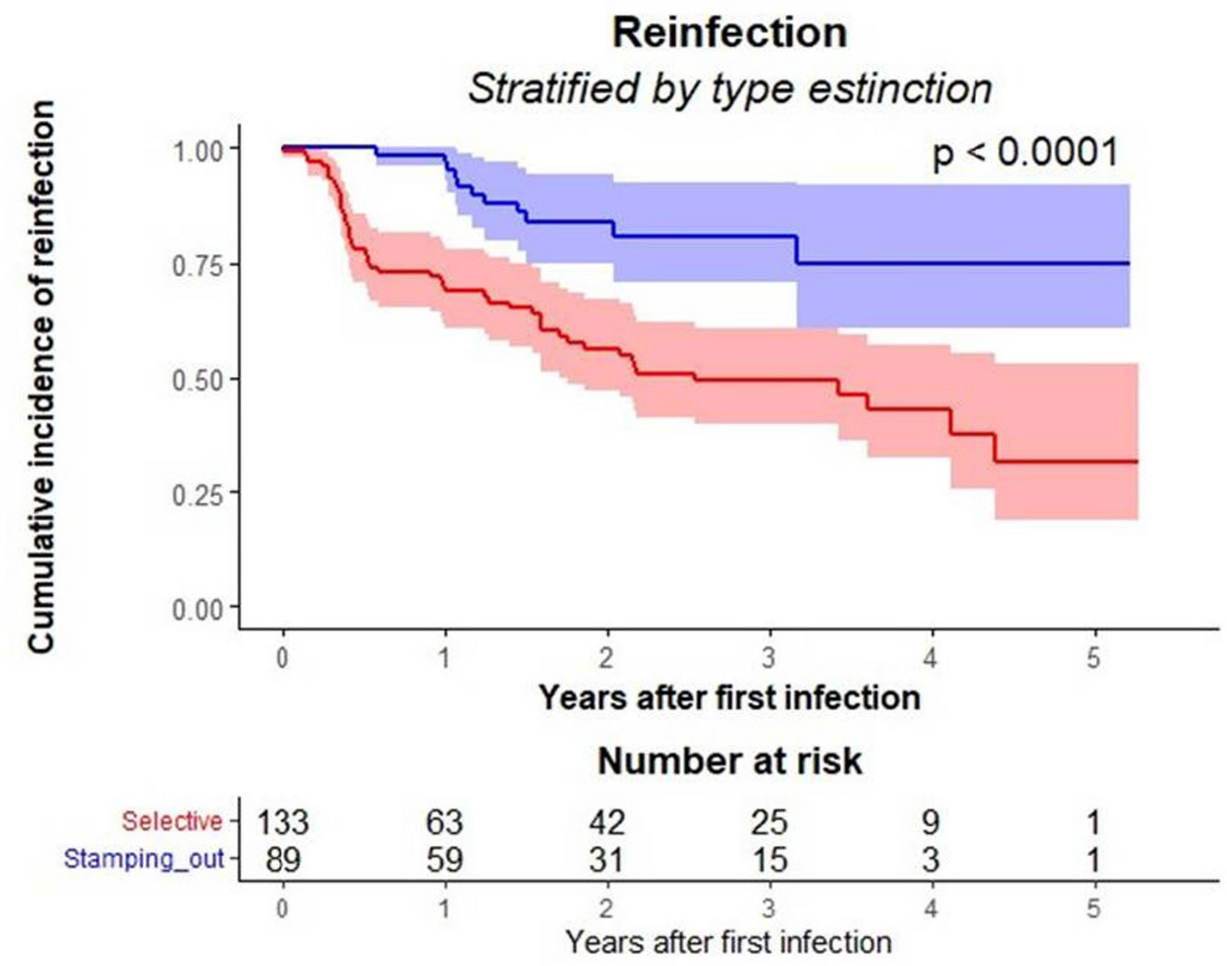
Cumulative incidence of reinfection stratified by eradication strategy. Kaplan–Meier survival curves showing time to reinfection in buffalo herds. The blue line represents herds controlled by stamping out, while the red line represents herds subjected to selective culling. The y-axis shows the cumulative probability of reinfection, corresponding to the proportion of reinfected farms over time. The x-axis denotes time in years since the first infection. A significantly higher reinfection rate was observed in herds subjected to selective culling compared with those managed by stamping-out (log-rank test, *p* < 0.0001). Shaded regions represent 95% confidence intervals. The number of farms at risk at each time point, reported below the plot, indicates herds still under observation and not yet reinfected or censored.

To further investigate differences in time to reinfection between eradication strategies, survival analysis was performed (Figure 3). Kaplan–Meier curves showed a significantly lower probability of reinfection over time in farms subjected to stamping out compared with those managed by selective culling (log-rank test, *p* < 0.0001). The steeper decline observed in the selective culling curve reflects a faster accumulation of reinfection events and a shorter disease-free period compared with stamping out. The number of farms at risk at each time point, reported below Figure 3, represents farms still under observation and not yet reinfected or censored.

Farms adopting stamping out showed a reinfection rate of 7.20% (95% CI: 3.6–12.9) whereas farms using selective culling had a reinfection rate of 27.06% (95% CI: 20.3–35.3). The median time to reinfection among herds subjected to stamping out was 2.5 years (95% CI: 1.8–3.1).

The log-rank test demonstrated a statistically significant difference (*p* < 0.0001), with a lower reinfection probability in farms subjected to stamping out.

The variable calculated based buffalo density in the municipalities where the outbreaks occurred was not significantly associated with reinfection (*p* = 0.12), with similar standard deviation values between the two groups (328 ± 164 *vs* 361 ± 134). Conversely, a statistically significant difference was observed in the number of buffaloes present at outbreak onset. Farms with reinfection had a significantly higher mean number of buffaloes at outbreak onset (389 ± 261 *vs* 298 ± 200; *p* = 0.013). The survival curves showing time to reinfection in buffalo herds, calculated according to the eradication method, are shown in Figure 3. The log-rank test demonstrated a statistically significant difference (*p* < 0.0001) in reinfection probabilities between the two eradication strategies, with a lower reinfection probability in farms subjected to stamping out compared to those where selective culling was applied.

At the bottom of Figure 3, the number of farms at risk is shown, corresponding to the number of farms still under observation and not yet reinfected or censored at each time point. A steeper descent of the red line (selective culling) represents a faster accumulation of reinfection events, indicating a shorter time to reinfection, meaning a shorter disease-free time.

In the univariate Cox regression analysis, stamping out and herd size at the outbreak onset were significantly associated with the time to reinfection, as shown in Table 2.

**Table 2:** Univariate Cox regression analysis of factors associated with time to reinfection.

Covariate	HR (95% CI)	*p* value
Stamping out	0.2 (0.1–0.5)	<0.001
Co-infection with *M. bovis*	0.9 (0.4–2.1)	0.9
Buffalo density per municipalities^a^	1 (0.9–1.1)	0.2
Buffalo number at the outbreak onset^+^	1.1 (1.1–1.2)	<0.001

a Buffalo density: hazard ratio calculated per increase of 200 buffaloes/km^2^ at the municipal level. Buffalo number at outbreak onset: hazard ratio calculated per increase of 50 animals. HR: Hazard Ratio per specified increment.

**Figure 4:**
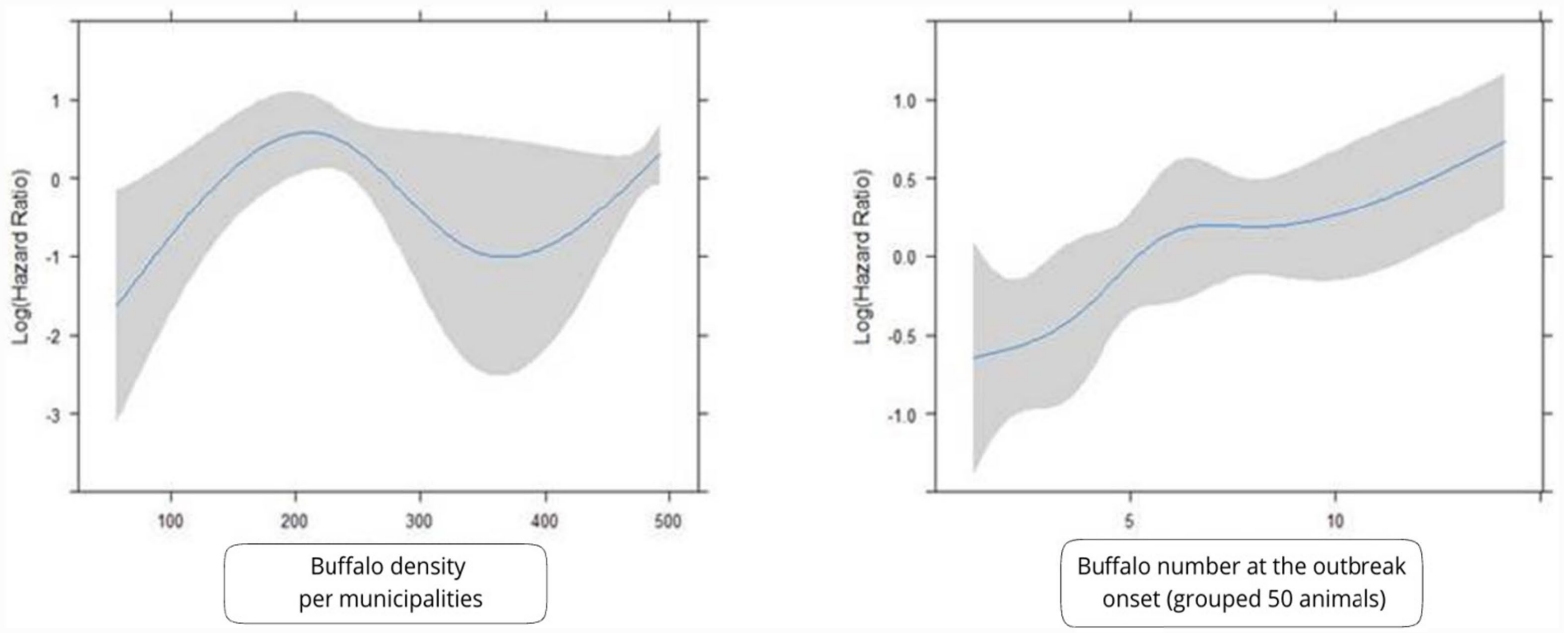
Restricted cubic spline analysis of the association between buffalo density at the municipal level (left) and herd size at outbreak onset (right) with reinfection risk. Shaded areas represent 95% confidence intervals.

The restricted cubic spline model indicated a significant non-linearity in the relationship between buffalo density at the municipality level and reinfection risk (*p* = 0.04), with a sharp increase in risk observed beyond a threshold of 200 animals/km^2^ (Figure 4), supporting the use of this cutoff value for risk stratification.

Based on this threshold, the best-fitting multivariable Cox proportional hazards model (lowest BIC = 582.3) included four covariates: eradication strategy (stamping out), buffalo density at the municipal level, herd size at outbreak onset, and coinfection with *M. bovis* (Table 3).

**Table 3:** Final Cox regression model (*N* = 222, events = 65).

Covariate	HR	*p*-value	95%CI
Buffalo density in municipalities^a^ (>200 animals/km^2^)	6.6	0.001	2.01–22.004
Buffalo number at the outbreak onset^+^	1.1	2.60 × 10^−7^	1.08–1.1
Coinfection with *M. bovis*	0.7	0.5	0.3–1.7
Stamping out	0.2	2.23 × 10^−6^	0.10–0.3

a Buffalo density in municipalities dichotomized as >200 vs ≤200 animals/km^2^. Buffalo number at outbreak onset: hazard ratio per increase of 50 animals.

Specifically, in the final Cox regression model (Table 3), buffalo density above 200 animals/km^2^ was associated with a 6.6-fold increase in reinfection risk (HR = 6.6; 95% CI: 2.01–22.00; *p* < 0.001), while herd size at outbreak onset showed a significant positive association with reinfection risk, with a hazard ratio of 1.1 for every additional 50 animals (*p* < 0.001). Coinfection with *M. bovis* was not significantly associated with reinfection (HR = 0.7; *p* = 0.5). In contrast, stamping out exerted a strong protective effect against reinfection (HR = 0.2; 5% CI: 0.10–0.30; *p* < 0.001).

Overall, 84% of the investigated outbreaks (*n* = 222) occurred in municipalities characterized by high buffalo density. Table 4 summarizes the municipalities identified as high-risk areas, including Cancello ed. Arnone, Castel Volturno, Santa Maria La Fossa, Baia e Latina, and Grazzanise, where buffalo density ranged from 233 to 492 animals/km^2^.

**Table 4:** Municipalities housing >200 buffalo/km^2^ in 2021 and brucellosis cases recorded since 2016.

Municipality	No. of buffaloes	No. of buffalo farms	Brucellosis prevalence in 2021	Brucellosis incidence in 2021	Municipality extension in km^2^	Density of buffaloes
Castel Volturno	16,833	111	49.4	34.8	72.2	233.0
Baia e Latina	5,889	25	0	0	24.5	240.3
Santa Maria La Fossa	9,973	39	20	15	29.5	337.8
Pastorano	5,264	8	0	0	13.8	380.1
Grazzanise	22,562	112	23.9	19.7	46.9	480.1
Cancello ed. Arnone	24,231	103	30.7	19.7	49.2	492.3

The density of buffaloes in municipalities, especially if it is greater than 200 animals/km^2^, shows a strong correlation with the risk of reinfection. The hazard ratio is estimated at 6.6 (*p* < 0.001), indicating a significantly increased risk of reinfection compared to areas with a lower buffalo density. The number of buffaloes housed on the farm at the beginning of the outbreak also shows a statistically significant correlation with the risk of reinfection. For every 50 additional buffaloes on the farm, the risk ratio increases by 1.1 (*p* < 0.001). Coinfection with *M. bovis,* the etiological agent of bovine tuberculosis (TB), shows a hazard ratio of 0.7 (*p* = 0.5), which indicates no statistically significant correlation with the risk of reinfection with *B. abortus*. The variable “stamping out” shows a strong protective effect against reinfection. The hazard ratio is estimated at 0.2 (*p* < 0.001), which indicates a significantly reduced risk of reinfection. Of the total number of outbreaks investigated (*n* = 222), 84% were observed in municipalities characterized by a high density of buffalo population. Table 4 shows the municipalities identified as high-risk areas along with the corresponding number of animals per square kilometer (population density) calculated for each municipality. The municipalities with a high risk of reinfection include Cancello ed. Arnone with a population density of 492 animals/km^2^, Castel Volturno with 233 animals/km^2^, Santa Maria La Fossa with 337 animals/km^2^, Baia e Latina with 240 animals/km^2^, and Grazzanise with 480 animals/km^2^.

Table 5 shows the epidemiological pattern observed in the period 2016–2024, including the effects of the Campania Region Decree 2022. After the peak of stamping out operations in 2021, the buffalo population decreased in the following 2 years and then started growing again until reaching the peak of the entire observation period.

**Table 5:** Epidemiological pattern in the period time 2016–2024 (VETINFO).

Year	Number of reared buffaloes	Number of positive heads	Number of culled heads	Percentage of culled buffaloes	Number of stamping out operations
2016	180,678	2,728	2,516	1.39	9
2017	164,138	3.797	3,373	2.05	10
2018	185,698	5.728	5,365	2.88	15
2019	182,611	8,766	9,098	4.98	17
2020	180,000	11,930	10,863	6.03	14
2021	180,422	8,943	8,216	4.55	30
2022	175,143	9.393	8,858	5.05	12
2023	176,532	6,164	6,096	3.45	10
2024	186,402	3,016	3,116	1.67	8

In the frame of human health, although no food-borne human case occurred in the EU in 2023 (EFSA and ECDC (European Food Safety Authority and European Centre for Disease Prevention and Control), 2024) (Table 6), sporadic food-borne brucellosis outbreaks continue to occur worldwide, mainly among consumers of unpasteurized dairy products worldwide, particularly cheese (Islam et al., 2023). Within the EU, a food-borne brucellosis outbreak due to raw milk was reported in 2019 (Table 6), while 16 food-borne outbreaks caused by *Brucella* spp. were documented between 2005 and 2018, four of which were linked to cheese consumption (EFSA and ECDC (European Food Safety Authority and European Centre for Disease Prevention and Control), 2017, 2018, 2019, 2020, 2021, 2022, 2023; ECDC Atlas). Ensuring the DFS of production zones is therefore crucial not only for safeguarding the reputation and quality of PDO dairy products, but also for protecting farmers and dairy workers, who remain at occupational risk of infection.

**Table 6:** Human brucellosis: number of reported cases in Italy and food-borne outbreaks in EU/EAA (EFSA and ECDC, One Health Zoonoses Reports, years 2016–2023; ECDC Atlas).

Year	Number of human cases reported in Italy	Notification rate (*N*/100,000) in Italy	Number of food-borne outbreaks reported in the EU/EEA	Number of human cases with infection acquired in the EU/total human cases reported in the EU	Notification rate (*N*/100,000) in the EU
2016	211	0.35	0	194/516	0.12
2017	99	0.16	1	148/378	0.09
2018	94	0.16	0	133/332	0.08
2019	49	0.08	1 (raw milk)	126/309	0.06
2020	18	0.03	1	68/132	0.03
2021	32	0.05	1	76/162	0.03
2022	48	0.08	0	66/226	0.05
2023	74	0.13	0	87/259	0.06
2024	33	0.06	–	–	–

For the epidemiological investigation of human isolates, it is strongly recommended to perform serotyping and genotyping using the core genome multilocus sequence typing (cgMLST) approach based on whole genome sequencing (WGS). This should be complemented by the analysis of specific single nucleotide polymorphisms (SNPs) obtained by mapping raw sequencing reads to the reference strain genome. The construction of minimum spanning trees can then be used to perform phylogenetic analyses and correlate samples of animal and environmental origin, with the aim of identifying the source of infection (Garofolo et al., 2017; Janowicz et al., 2018).

## 4 Discussion

Beyond its veterinary relevance, brucellosis still poses a risk to public health and food safety. Although no foodborne human cases of brucellosis were reported in Italy in 2023 (EFSA and ECDC (European Food Safety Authority and European Centre for Disease Prevention and Control), 2024), sporadic outbreaks continue to arise among consumers of unpasteurized dairy products, especially artisanal cheeses (Islam et al., 2023). These products need to maintain their microbiological safety to preserve their international prestige. Water buffalo brucellosis remains a persistent endemic challenge in the province of Caserta. Although overall incidence has decreased over the last three decades, reinfections continue to occur, especially in areas of high buffalo density and large herd size (Mazzeo et al., 2023). In fact, larger herds and densely populated areas facilitate disease persistence and reinfection (Coelho et al., 2015; Dadar et al., 2021).

In this context, the national buffalo traceability system currently implemented in Italy represents an important complementary tool for disease control. Developed and validated through a Ministry of Health project and subsequently made mandatory nationwide, this online platform allows continuous monitoring of buffalo movements, milk production, and dairy processing along the entire supply chain (Cappelli et al., 2021). In high-density production areas such as the province of Caserta, traceability supports food safety, facilitates official controls, and enhances the management of disease outbreaks; nonetheless, it cannot fully offset the risk of reinfection, which may be amplified in extensive and pasture-based production systems due to increased animal-to-animal contact favoring bacterial transmission (Mazzeo et al., 2025b).

Following the strategies implemented for its eradication, the incidence of brucellosis has steadily but slowly decreased over time (Mazzeo et al., 2025b).

Within this framework, Italy, and particularly the province of Caserta, can be regarded as a paradigmatic example of a high-density buffalo production system where brucellosis persists despite the long-term implementation of sanitary and regulatory measures (Tittarelli et al., 2015; Mazzeo et al., 2025b). Although national and regional control frameworks are well established, comparatively limited attention has been devoted to the field-based comparative evaluation of different eradication strategies, both in Italy and in similar endemic settings worldwide (Dean et al., 2012).

One of the reasons for the persistent difficulty in achieving complete eradication of brucellosis is the detection of infections in herds that have experienced reinfection. The present findings are consistent with evidence from Italy and comparable endemic territories, indicating that whole-herd depopulation (stamping out), when followed by thorough cleaning, disinfection, and delayed restocking, is associated with a lower probability of recurrence than selective culling in high-density settings [Coelho et al., 2015; Dadar et al., 2021; World Organisation for Animal Health (WOAH), 2024]. Conversely, selective culling alone appears insufficient in high-density settings, as residual carriers or latent infections may perpetuate the pathogen cycle within and between farms. This supports the European SANCO/6095/2009 (2009) recommendations emphasizing that total depopulation should be prioritized in areas with high infection pressure or repeated outbreaks.

The Cox model identified two primary risk determinants: (i) large herd size at outbreak onset, which correlates with higher transmission potential; (ii) buffalo density exceeding 200 animals/km^2^ which increases reinfection risk by approximately sixfold. These established risk factors should inform targeted interventions and resource allocation in the context of updated One Health objectives. The increased prevalence of brucellosis in large herds has been reported to be associated with several factors, such as a higher number of tested animals in larger herds. This would mean that the probability of detecting at least one seropositive animal is greater or that the higher number of animals increases the possibility of spreading the disease through direct and indirect contact. Other crucial factors that emerged include the timely detection of clinical signs (e.g., abortion and retained placenta) and effective disinfection of farm premises. Clinical signs are often underestimated, since large herds may require the presence of several workers who handle more than one group of animals during the day. In terms of disinfection and cleaning, cost and time influence these risk factors. For disinfection and cleaning procedures to be effective in paddocks with a significant number of animals, complex and costly coordinated procedures are required, consisting of moving groups of animals to temporary paddocks, mucking out, and cleaning and disinfecting with approved product (respecting exposure times and quantity/m^2^). The low prevalence of brucellosis in small herds could also be related to the management of the herd and/or farm. In smaller farms, cleaning, disinfection, and manure removal are easier and less time-consuming for the farmer. Farmers with small herds find it easier to respect the birthing time and usually keep the dams away from the herd during parturition. This measure is very important in the event of abortions to avoid contamination of the farm environment. In these small herds, new animals are usually replaced with animals born on the same farm, avoiding commercial trade. The absence of a high proportion of animal purchases therefore reduces the risk of introducing infections (Coelho et al., 2015). The buffalo density in a single municipality (>200 animals/km^2^) is crucial for the reinfection risk of Caserta herds. In fact, a large part of the buffalo population is in four municipalities (Cancello ed. Arnone, Castel Volturno, Grazzanise, and Santa Maria La Fossa) characterized by a complex hydrographic network that includes many livestock farms (Mazzeo et al., 2023). Other authors have pointed out that maintaining a high population density of animals in a small area facilitates the transmission of the disease, which may be an important factor in its spread (Van Seventer and Hochberg, 2016; Dadar et al., 2021).

In addition, some behavioral characteristics of buffaloes, e.g., the long stay in dung, favor exposure to *B. abortus*, which can survive under favorable humidity and temperature conditions (Wray, 1975; Borriello et al., 2013). The overall health status of the herd may also influence susceptibility to brucellosis. We included co-infection with *M. bovis* as a covariate because the co-circulation of *M. bovis* and *Brucella* spp. in buffalo populations has been documented in field studies and may influence host susceptibility and transmission dynamics (da Silva et al., 2014; Carneiro et al., 2019). Incorporating this variable allowed us to explore whether areas or farms with evidence of bovine tuberculosis differed in brucellosis reinfection risk. However, in our case, the association between tuberculosis and reinfection with *B. abortus* was not significant, showing an inverse trend. This may reflect differences in management practices adopted to mitigate the prolonged environmental persistence of *M. bovis*. Moreover, evidence from epidemiological modeling suggests that the interaction between *Brucella* spp. and *M. bovis* may have contrasting outcomes at different scales. While bovine tuberculosis can facilitate brucellosis infection at the individual level, *Brucella* infection appears to exert a strong negative effect on *M. bovis* at the population level, reducing its endemic prevalence and basic reproduction number (Gorsich et al., 2018). Our findings align with Schwarz et al. (2021), who reported that brucellosis and tuberculosis show distinct but overlapping spatiotemporal clusters in buffalo herds, and highlight the need to consider dual-pathogen dynamics in endemic settings.

Despite the widespread adoption of stamping out and selective culling as cornerstone approaches in brucellosis control programs, direct field-based comparative evidence on their effectiveness and impact on reinfection risk remains limited, particularly in water buffalo production systems (Dean et al., 2012). This lack of data-driven comparative studies represents a significant knowledge gap that constrains fully evidence-based policy-making in endemic areas.

The results of our study show that stamping out of the infected epidemiological unit is the most important and efficient measure to eradicate brucellosis, provided that the infected animals are immediately removed from the farm and the restocking is carried out in a way that prevents or limits the introduction of the infection (SANCO/6095/2009, 2009). In the Campania region, very strict structural and biosecurity requirements are justified based on the high incidence and prevalence of brucellosis in the buffalo population (Campania Region, 2022). In addition to the strictly controlled and reinforced biosecurity measures required by national and regional laws, the practices of thorough cleaning and disinfection of the premises and a void period before restocking are certainly successful in preventing reinfection (SANCO/6095/2009, 2009). Education and training of farmers and farm workers play a key role in improving compliance with biosecurity measures and in promoting early recognition of clinical signs, particularly in settings where traditional practices may conflict with disease control strategies (Plumb et al., 2013).

Disseminating clear information on the economic and sanitary impact of brucellosis on the livestock sector and PDO dairy production may further enhance farmer engagement and accountability, contributing not only to the control of brucellosis but also to the prevention of other zoonotic and foodborne pathogens, such as *Salmonella* spp., including *S.* Umbilo, which caused a severe multi-country outbreak in 2024 (Mazzeo et al., 2025a). In water buffalo, the RB51 vaccine may be used in high-prevalence areas since it does not interfere with routine serological tests and allows discrimination between vaccinated and infected animals. In contrast, the Rev.1 vaccine widely used in small ruminants induces an antibody response that cannot be distinguished from field infection using standard serological assays, as demonstrated by Katsiolis et al. (2022), thereby creating the risk of misclassifying vaccinated animals as infected. This has significant epidemiological and commercial implications, as it may lead to unnecessary culling in compulsory eradication contexts. In Italy, the use of Rev.1 is not authorized, since national control strategies are based on eradication and selective culling, whereas RB51 may be considered only in “cluster municipalities,” where high prevalence and herd density require extraordinary measures to reduce infection pressure without compromising diagnostic accuracy (Katsiolis et al., 2022; Tittarelli et al., 2015; Mazzeo et al., 2025b).

In parallel, human brucellosis cases in Italy showed a constant decline between 2016 and 2020. According to ECDC surveillance data, Italy reported up to 211 human brucellosis cases in 2016 (0.35 per 100,000 inhabitants) [EFSA and ECDC (European Food Safety Authority and European Centre for Disease Prevention and Control), 2017], decreasing to 32 cases reported in 2021 (0.05 per 100,000 inhabitants) reflecting the progressive expansion of DFS zones and the effectiveness of eradication programs in reducing zoonotic transmission. However, Italy still reports some of the highest in the European Union, due to the persistent endemic circulation of *Brucella* spp. in southern regions, namely Campania, Puglia, and Sicily (EFSA and ECDC (European Food Safety Authority and European Centre for Disease Prevention and Control), 2022), supporting the need for an integrated One Health perspective (Mazzeo et al., 2025b). The discrepancy between the overall number of reported human cases and the relatively low number of foodborne outbreaks at EU level suggests that brucellosis in Italy remains predominantly an occupational disease.

Therefore, continuous monitoring of brucellosis outbreaks in buffalo farms and of human cases among occupationally exposed populations, together with the analysis of their temporal dynamics in endemic areas, is essential to achieve and maintain DFS without vaccination. Achieving DFS certification would also enhance the international competitiveness of PDO and other traditional buffalo dairy products, facilitating access to markets that require stringent animal health guarantees, such as China, which also mandates DFS for TB and Johne’s disease (Mazzeo et al., 2024), as well as countries that prohibit vaccination altogether (e.g., Brazil).

## 5 Conclusion

In endemic areas, stamping out combined with adequate restocking and reinforced biosecurity remains the most effective strategy. Key determinants of reinfection in the present study were herd size and buffalo density, with densities above 200 animals/km^2^ markedly increasing the risk. Therefore, targeted action is needed in densely populated areas, requiring coordinated regional programs that integrate biosecurity, surveillance, and risk stratification. Furthermore, collaboration between the veterinary and public health sectors is crucial for implementing the integrated One Health strategy for brucellosis. Importantly, maintaining a Disease-Free (DF) status without vaccination is a fundamental regulatory and economic requirement under Regulation (EU), 2016/429 (“Animal Health Law”), as this represents the highest certification level for unrestricted movement and trade of live animals and derived products within the EU and internationally. Vaccination would compromise this certification pathway due to potential interference with serological surveillance and the risk of masking infection, thereby limiting market access and hampering disease eradication programs.

## Data Availability

The datasets presented in this study can be found in online repositories. The names of the repository/repositories and accession number(s) can be found at: VETINFO, www.vetinfo.sanita.it.
